# Maternal Psychological Distress and Executive Functions Are Associated During Early Parenthood – A FinnBrain Birth Cohort Study

**DOI:** 10.3389/fpsyg.2021.719996

**Published:** 2021-10-22

**Authors:** Elisabeth Nordenswan, Kirby Deater-Deckard, Eeva-Leena Kataja, Mira Karrasch, Juho Pelto, Matti Laine, Hasse Karlsson, Linnea Karlsson, Riikka Korja

**Affiliations:** ^1^The FinnBrain Birth Cohort Study, Turku Brain and Mind Center, Department of Clinical Medicine, University of Turku, Turku, Finland; ^2^Department of Psychology, Åbo Akademi University, Turku, Finland; ^3^Department of Psychological and Brain Sciences, University of Massachusetts Amherst, Amherst, MA, United States; ^4^Department of Psychiatry, Department of Clinical Medicine, University of Turku and Turku University Hospital, Turku, Finland; ^5^Centre for Population Health Research, Turku University Hospital and University of Turku, Turku, Finland; ^6^Department of Psychology, University of Turku, Turku, Finland

**Keywords:** executive functioning, early parenthood, parenting, early childhood, psychological distress

## Abstract

Parental executive functioning (EF) and parenting behaviors can be affected by the multiple stressors that are often present during early parenthood. However, little is known about how commonly experienced psychological distress during early parenthood is associated with parental EF capacity. We explored the links between psychological distress and EFs in a general population sample of 150 Finnish birth cohort mothers with 2.5-year-old children. The symptoms of depression, anxiety, insomnia, and poor couple relationship adjustment were measured with the self-report questionnaires EPDS, SCL-90, AIS, and RDAS. EFs were assessed with five computerized Cogstate tasks. When the psychological distress measures were added to a hierarchical regression analysis as continuous variables, no significant single or additive associations with EFs were found. When the distress measures were dichotomized to compare symptoms below/above cutoffs indicating clinically elevated levels, single distress domains remained as non-significant predictors, but a cumulative risk index of the number of concurrent clinically elevated distress domains was significantly associated with EFs. Thus, mothers with a higher number of concurrent clinically elevated psychological distress domains (i.e., depression, anxiety, insomnia, and poor couple relationship adjustment) tended to have lower EFs. This association is possibly bi-directional – clinically elevated distress within several domains could have a cumulative, depleting effect on maternal EF capacity, but a lower EF capacity could also increase the vulnerability for experienced distress within several concurrent domains. Longitudinal studies are needed to clarify potential causal links between stressors and EF.

## Introduction

Decades of research have provided detailed knowledge about how parental caregiving behavior plays a key role in child neural, affective, and cognitive development (see, e.g., Tottenham, 2020). A detailed understanding of specific societal, familial, and individual components of parenting is needed to support optimal child development. At the level of the individual parent, a growing number of recent studies have identified parental executive functioning (EF) to be one of the components that shape parenting (Bridgett et al., 2015; Crandall et al., 2015). EF capacity is negatively associated with stress (Diamond, 2013; Shields et al., 2016). However, little is known about how maternal EF capacity is related to various domains of psychological distress commonly experienced by mothers during early parenthood. Studies exploring the role of EFs in the context of early parenthood are needed to aid healthcare providers and policy makers striving to identify families in need of supportive interventions.

### Executive Functions, Parenting, and Psychological Distress

The foundation of EFs is thought to consist of three closely related core functions. *Working memory updating* refers to the ability to process information that is relevant for the task at hand, by monitoring and coding incoming information and by replacing no longer relevant information with more relevant, newer information. *Inhibitory control* enables the suppression of prepotent responses. *Set-shifting* refers to the ability to flexibly shift between multiple mental sets or tasks (Miyake et al., 2000). Higher-order goal-directed behavior like planning, reasoning, and problem solving is thought to build upon these core functions. At the neural level, EF domains are related to frontal-cingulate-parietal-subcortical networks, where the prefrontal cortex plays a central role (Friedman and Miyake, 2017). EF skills are not only relevant for cognitive functions; they are central for a wide array of different aspects of human development, health, and wellbeing *via* their roles in the cognitive and emotional regulation of our thoughts, behaviors, and even physiology (Diamond, 2013; Bridgett et al., 2015).

Researchers have recently started to uncover the complex links between maternal EFs and parenting, finding EFs to be a fundamental component of mothering alongside emotion and stress regulation systems (Barrett and Fleming, 2011). A sufficient EF capacity allows mothers to maintain and manipulate information in working memory in order to plan and carry out childcare, and to flexibly shift and focus their attention in sometimes highly stimulating environments. Better EFs are generally related to sensitive, involved parenting, while a lower EF capacity is related to harsher parenting and an increased risk of engaging in child maltreatment (Bridgett et al., 2015; Crandall et al., 2015).

EFs are generally negatively associated with common adversities and symptoms like sadness, sleep deprivation, and lack of social support (Diamond, 2013). Acute stressors that occur and cease relatively quickly (e.g., the trier social stress test), have been shown to impair the central EF capacities of working memory and set-shifting, while simultaneously having an enhancing effect on response inhibition (see Shields et al., 2016, for a review of acute stress and EFs among primarily young adults). The effect of chronic stressors that persist over time (e.g., poverty, unemployment, and inadequate housing) on EF has not been as extensively studied. There are a few findings of associations between chronic stress and poorer EF performance among young adults (Orem et al., 2008; Tomeo, 2014), and chronic stress occurring over the life span has been linked to structural changes in brain regions that are central for EFs (Shields and Slavich, 2017). This suggests that besides acute stress, chronic stress can also have a deleterious influence on EF capacity.

The transition to parenthood and the period of early parenthood are often joyous and gratifying as well as challenging and stressful. Young children’s caregiving needs can be strenuous, influencing parents’ wellbeing negatively and resulting in depression and anxiety, sleep disturbance, and strained partner relationships (Nelson et al., 2014; Canário and Figueiredo, 2017). Given that caregiving quality has been linked to maternal EF capacity, EFs are negatively affected by stressors, and several stressors are common in early parenthood, the question arises whether psychological distress common in early parenthood could be related to lower maternal EFs. In the present study, we explored whether maternal psychological distress, including here symptoms of depression, anxiety, insomnia, and poor couple relationship quality, is associated with maternal EFs during early parenthood.

### Depression, Anxiety, and Executive Functions

Depression and anxiety are common in the general population: Depressive disorders have a lifetime prevalence estimate of 20.8% in western countries, while the corresponding estimate for anxiety disorders is 28.8% (Kessler et al., 2005). Depression and anxiety are also common during early parenthood, i.e., the first several years of parenthood. During the first year after delivery, postpartum depression has a global prevalence of 17.7% (Hahn-Holbrook et al., 2018), and 8.5% of postpartum mothers experience one or more anxiety disorders (Goodman et al., 2016). While postpartum depression typically develops during the first few months of an infant’s life and often remits within a few months, some mothers develop a chronicity. Up to one-third of the mothers who become depressed during the early postpartum period still suffer from depressive symptoms at 2years after delivery (Goodman, 2004). There are also reports of depression and anxiety symptom trajectories stretching up to 2.5years into the postpartum period, suggesting that depression and anxiety symptoms related to the transition to parenthood can last beyond the early postpartum infancy period into toddlerhood (Canário and Figueiredo, 2017). Depression and anxiety can coexist or occur independently. As they frequently coexist during early parenthood, they are often jointly assessed (Canário and Figueiredo, 2017).

Both depression and anxiety have been linked to impairments in adult EF capacity (see, e.g., Castaneda et al., 2008; Snyder, 2013). Snyder et al. (2015) state that there is a wealth of evidence indicating that adults suffering from different psychopathologies, like depression and anxiety, perform worse on EF tasks than healthy controls. There are reports of links between maternal depression/anxiety symptoms and poorer EFs during early parenthood, starting as early as in the prenatal period. For example, when examining partly the same participants as the ones who took part in this study, Kataja et al. (2017) found that mothers who during pregnancy reported a high amount of depressive and pregnancy related anxiety symptoms made significantly more errors in an EF task compared to mothers who reported low symptom levels. This highlights the importance of further investigating the links between maternal depression/anxiety and EF in early parenthood.

### Insomnia and Executive Functions

Sleep disturbances (e.g., nighttime awakenings and shorter sleep duration) are common among mothers of young children. Approximately 30% of mothers with children younger than 3years feel that their daytime functioning is affected by their child’s sleep pattern (Mindell et al., 2015). Sleep patterns have been linked to maternal EFs and parenting in early parenthood. Chary et al. (2020) found that for mothers of typically developing 2.5-year-old children, both maternal sleep activity (i.e., restlessness and night waking) and sleep duration came together with EFs to statistically interact in predicting the degree of harsh parenting. Indeed, insomnia and EFs are negatively associated across adulthood; adults diagnosed with insomnia perform poorer on EF tasks than healthy controls (Ballesio et al., 2019a). Sleep deprivation has been found to trigger brain activity changes, which predict severity of impairment in working memory tasks (Krause et al., 2017).

### Couple Relationship Quality and Executive Functions

The transition to parenthood can be linked to lowered couple relationship quality. The infant’s demands of attention and care reduce the amount of time parents can spend by themselves or together, and parents are required to cope with potentially stressful caregiving situations (Kluwer, 2010). Kluwer also reports a small but reliable decrease in couple relationship quality at this family life stage in western countries, with approximately half of the couples experiencing these negative changes. Furthermore, longitudinal studies suggest that this decline in couple relationship quality can persist over time, affecting couples for several years after childbirth (Kluwer, 2010). Although the effects of poor couple relationship quality on EFs have not been extensively studied, an association is likely, as poor couple relationship quality is a well-established stressor that can have wide-ranging negative health effects (Cohen et al., 2019), and stressors are known to deplete adult EF capacity (Diamond, 2013). Studies of couples’ interactions including physiological assessments have linked marital conflict to health-related physiological mechanisms, like alterations in stress hormones, cardiovascular activity, and dysregulation of immune function (Robles and Kiecolt-Glaser, 2003). Similar physiological mechanisms are described in the literature linking chronic stress to structural changes in brain regions that are central for EFs (Shields and Slavich, 2017). This provides further evidence for a likely association between poor couple relationship quality and a depleted EF capacity.

### Cumulative Effects of Psychological Distress

When studying the associations between psychological distress and EFs, it is important to besides individual effects of single distress domains also consider potential additive/cumulative effects. Exposure to multiple risk factors predicts more severe adverse consequences in comparison to single risk factor exposure, and multiple combinations of different stressors can account for the variability in the outcome variable of interest, such as parental EF (Evans et al., 2013). An additive risk model examines to what extent each risk factor contributes both independently and together with other risk factors to the outcome variable of interest. In contrast, a cumulative risk model focuses on the number of risk factors present, exploring whether a varying number of concurrent risk factors is associated with the outcome variable (Burchinal et al., 2000; Jones et al., 2002).

### The Current Study

The associations between lower maternal EFs and poorer caregiving quality described above underscore the need for a better understanding of the interplay between common psychological distress domains during early parenthood and maternal EFs. In this study, we examined how four closely related domains of distress (self-reported symptoms of depression, anxiety, and insomnia, along with poor couple relationship quality) were related to EFs in a general population sample of mothers with 2.5-year-old children. We chose to focus on mothers of toddlers, as toddlers’ have rapidly changing cognitive, language, and motor development and actively explore the physical world (Payne and Isaacs, 2017; Madigan et al., 2019), while simultaneously requiring large amounts of external self-regulatory support from caregivers (Bridgett et al., 2015). The effects of maternal EF capacity on caregiving behavior are therefore likely to be particularly pronounced during this period, because toddlers’ demands on parents are so substantial.

Based on the literature presented above, we hypothesized that psychological distress would be associated with lower EFs. Furthermore, we expected to find cumulative effects, so that the psychological distress/EF associations would become stronger when simultaneously considering several domains, in comparison with the associations between single psychological distress domains and EFs. To the best of our knowledge, no previous study has explored how these psychological distress symptoms that are common during early parenthood, are related to maternal EF in a general population sample, while considering both single and cumulative effects of distress domains.

## Materials and Methods

### Participants

Participants (*N*=150) were recruited from the FinnBrain Birth Cohort, which explores child development and parenting (*N*=4,000 families; Karlsson et al., 2018). This study’s participants’ mean age at 2.5years postpartum was 35.08years (*SD*=4.47years, range=24.05–46.18years). One-third of the mothers (33.3%) were primiparous, 49.3% had two children, 15.3% had three children, and 2.0% had four children. Additionally, 8.7% of the mothers were pregnant during the collection of this study’s main variables, i.e., at 2.5years postpartum. Information about maternal education level, occupation, and income level was collected when the pregnant mothers were recruited to the main cohort, approximately 3years prior to the collection of this study’s main variables of interest. At this time, almost half of the participants (48.7%) had a university level education, 32.0% had a polytechnics education, while 19.3% had a high school/vocational education (<12years). A majority of the participants (80%) were employed, while few were stay-at-home mothers (7.3%), students (4.0%), unemployed (5.3%), or otherwise occupied (3.4%). After taxes, 30.2% of the participants had a total monthly income of 1,500€ or less, 59.1% had an income between 1,501€ and 2,500€, 8.7% had an income between 2,501 and 3,500€, and 2% had an income over 3,500€.

### Procedure

The Joint Ethics Committee of Turku University Hospital and University of Turku gave ethical approval for this study. Written informed consent was required before participation. The main FinnBrain cohort (*N*=4,000 families) was recruited in southwest Finland between 2011 and 2015. Families joined the study when attending free-of-charge pregnancy ultrasound scans at maternal welfare clinics during gestational week 12, with coverage of contacted families close to 100% in the population. Sufficient knowledge of Finnish or Swedish and a normal ultrasound screening result were required for participation (Karlsson et al., 2018).

The current study’s participants took part in a broader sub-study within FinnBrain, which explores maternal cognition and child self-regulation development. Mothers from the FinnBrain Birth Cohort were from 2012 to 2013 randomly selected for recruitment to this sub-study. Exclusion criteria were self-reported psychiatric or neurologic illness and insufficient Finnish language skills. The sub-study’s first study visit was conducted during pregnancy, and mothers who had attended this first visit were invited back for follow-up study visits at 1year and 2.5years after delivery. At the recruitment of participants for the 2.5-year study visit, the recruitment list was expanded with mothers whose children had participated in a separate FinnBrain study visit. In total, 341 mothers were contacted during the recruitment of participants to the 2.5-year study visit. Of these, 290 were reached and informed about the study (85% of contacted). Of these, 211 booked a visit (76.2% of reached). Of these, 198 completed the visit (89.6% of booked). Of the 198 mothers who completed the 2.5-year study visit, 150 had also filled out a couple relationship questionnaire sent home to the whole cohort at 2years after delivery, reflecting that they were in a relationship at that time. Only these mothers who completed the couple relationship questionnaire and participated in the 2.5-year study visit were included in the current study, because having valid couple relationship data was central for the study question.

The study visit for mothers at 2.5years after delivery included (among other tasks) computerized EF measures, verbal intelligence tasks, and depression, anxiety, and insomnia questionnaires. The study visits were conducted by graduate students in quiet examination rooms at the University of Turku facilities. Questionnaires assessing current partner relationship quality had previously been filled out at home 6months earlier.

Of the 290 mothers who were reached during the 2.5-year study visit recruitment, the 198 who completed the study visit did not differ significantly from those who did not in terms of age, *t*(288)=−1.05, *p*=0.29, or education level [*X*^2^(2, *N*=290)=4.05, *p*=0.13]. Of the mothers with completed study visits, there was no significant difference in education level for the mothers in/not in a relationship [*X*^2^(2, *N*=197)=3.43, *p*=0.18], but the 150 who were in a relationship were significantly older than those who were not [*t*(196)=3.21, *p*=0.00]. The current study’s sample of 150 mothers was on average significantly older than the remaining mothers in the whole FinnBrain cohort [*t*(3806)=4.40, *p*<0.001]. At delivery, the participants in this study had a mean age of 31.9years, while the remaining mothers in the whole cohort had a mean age of 30.2years. The current study’s participants had also attained a significantly highly level of education than the remaining mothers in the whole cohort [*X*^2^(2, *N*=3,078)=26.19, *p*<0.001].

### Measures

#### Depression Symptoms

The Edinburgh Postnatal Depression Scale (EPDS; Cox et al., 1987) was used to measure depression symptoms. In this 10-item self-report questionnaire, participants report depression symptoms experienced during the past 2weeks using a 4-point Likert scale. The EPDS has been studied extensively and is seen as a valid measure of postnatal depression (Smith-Nielsen et al., 2018). The EPDS had good internal consistency in the current study’s sample (*α*=0.86). We employed the total EPDS sum score in our analyses, in which a higher value indicated more symptoms of depression. We also utilized a dichotomized EPDS variable, which was split according to the cutoff value of 11 or more, which is considered to indicate depression (Smith-Nielsen et al., 2018).

#### Anxiety Symptoms

Anxiety symptoms were measured with the anxiety subscale from the Symptom Checklist 90 (SCL-90; Derogatis et al., 1973). The SCL-90 anxiety subscale consists of 10 self-report items, assessing anxiety symptoms experienced during the past month. Items are rated on a 5-point Likert scale. One participant had one missing value on the SCL-90 anxiety subscale, which was imputed with the other item’s mean value. The SCL-90 had good internal consistency in our sample (*α*=0.78). The total SCL-90 anxiety subscale sum score was utilized in our analyses, in which a higher value reflects more anxiety symptoms. The SCL-90 cutoff score for moderate anxiety symptoms is 7.5, while the cutoff score for severe anxiety symptoms is 13.5 (Schmitz et al., 2000). In addition to using the continuous SCL-90 variable, we also analyzed a dichotomized variable that differentiated whether participants’ SCL-90 results were below/above the cutoff score of 7.5 points.

#### Insomnia Symptoms

The Athens Insomnia Scale (AIS; Soldatos et al., 2000) was used to assess insomnia symptoms. This 8-item self-report measure is designed for brief and easy quantification of sleep difficulty based on the International Classification of Diseases (ICD-10) criteria and has sound psychometric properties (Soldatos et al., 2003). The AIS had good internal consistency in our sample (*α*=0.80). We used the total score, ranging from 0 (absence of sleep-related problems) to 24 (the most severe degree of insomnia). A cutoff score of 6 or higher indicates insomnia (Soldatos et al., 2003). In addition to analyzing the continuous score, we also utilized a dichotomized AIS variable, which was split according to the cutoff value. One participant had a missing AIS item value, which was imputed with the other items’ mean value.

#### Couple Relationship Quality

The Revised Dyadic Adjustment Scale (RDAS; Busby et al., 1995) was used to measure self-reported partner relationship adjustment. The RDAS is widely used and has sound psychometric properties (Turliuc and Muraru, 2013). This 14-item questionnaire produces an overall marital adjustment score (range: 0–69). Higher scores indicate greater relationship satisfaction; lower scores indicate greater marital distress. A cutoff score of 47 or lower distinguishes distressed couples from non-distressed couples (Turliuc and Muraru, 2013). The RDAS had good internal consistency in our sample (*α*=0.86). We used a reversed overall adjustment score, so that a higher value equaled a worse couple relationship. A dichotomized RDAS variable was also utilized, which was split according to the cutoff value. Three participants had one missing RDAS item value. These were imputed with the subscales “other items” mean values.

#### Executive Functioning

EFs were measured with a composite score encompassing five Cogstate tasks. The Cogstate test battery includes computerized adaptations of standard neuropsychological tests (Nordenswan et al., 2020). As any EF task engages both general (EF) and task-specific cognitive processes, it is preferable to base EF assessment on multiple tasks (Friedman and Miyake, 2017). During the more extensive study visit for mothers that was conducted at 2.5years postpartum, the participants completed 12 Cogstate tasks on a laptop computer during approximately 1h. From this measurement, five tasks thought to measure EFs were selected for this study. In accordance with a previous factor analytic study, the task outcome variables thought to best capture EF-related variance were used. Within a sample of general population mothers from the FinnBrain Birth Cohort (Karlsson et al., 2018), Nordenswan et al. (2020) explored the intercorrelation between five Cogstate tasks that in previous studies had been labeled as EF/learning tasks; the Two Back Test (TWOB), the Set-Shifting Test (SETS), the Groton Maze Learning Test (GML), the Continuous Paired Associate Learning Test (CPAL), and the International Shopping List Test (ISL). Confirmatory factor analysis (CFA) demonstrated that a single-factor solution was a good fit for the five tasks. This prior evidence supports combining these tasks into an EF/learning sum score, allowing for more reliable assessments compared to the use of single tasks. Nordenswan et al. (2020) further examined whether the choice of indicator (i.e., score) for the three tasks that had multiple test rounds (i.e., GML, CPAL, and ISL) affected the single-factor model’s properties. Cogstate tasks yield multiple outcome variables, of which Cogstate recommend primary outcome measures as indicators that are optimal for detecting cognitive change. For tasks with multiple test rounds, the recommended primary outcome measure summarizes the test performance across all test rounds. Novel tasks are known to tap more into EFs than familiar tasks, but novel tasks also become more familiar with repeated practice within a test session. Therefore, Nordenswan et al. (2020) ran two separate CFA’s including different outcome variables from the same tasks. The first CFA model included the traditional sum scores test performance across all rounds for the tasks with multiple test rounds, i.e., GML, CPAL, and ISL. Based on the factor loadings (which were especially high for GML and CPAL, and particularly low for SETS), this model was interpreted to tap primarily into learning. The second CFA model included the first test round for GML, CPAL, and ISL, i.e., the period where task novelty could exert its effect. The more even factor loadings of this model suggested that it had a stronger executive function component. Based on these findings, we utilized the task outcome measures from the second CFA model by Nordenswan et al. (2020) to optimize the specificity of our EF composite. For TWOB, the Cogstate recommended arcsine transformation of the square root of the proportion of correct responses was chosen as the outcome variable. The same outcome variable was also chosen for SETS, as it was better distributed than the number of errors, which is the recommended outcome variable. The two measures in SETS capture the same variance, which is demonstrated by their complete correlation (*r_s_*=−1.00, *p*=0.00). For GML and CPAL, the number of errors from the first test round (i.e., the round after the first learning trial) was chosen as outcome variables. For ISL, the number of correct responses from the first round was chosen as outcome variable. The GML and CPAL outcome variables were reversed, so that a higher value equaled a better result for all Cogstate tasks. The task scores were standardized, combined into an EF mean score, and re-standardized. The Cogstate tasks are described in more detail below. See Nordenswan et al. (2020) for more details on the EF composite. Based on the CFA’s factor loadings that are presented in Nordenswan et al. (2020), the EF composite utilized in this study is likely to have a large working memory updating component, which primarily captures visuospatial capacities while also including verbal abilities. The composite also taps into set-shifting but does not include a separate measure of inhibitory control.

##### The Two Back Test

This working memory task is based on the n-back paradigm. The participant is to decide whether the playing card shown at the center of the screen is identical to the one presented two cards previously. The task terminates after 32 correct responses. Outcome variable: the arcsine transformation of the square root of the proportion of correct responses.

##### The Set-Shifting Test

This task is similar to the Wisconsin Card Sorting Test, a widely used EF task considered to tap into set-shifting ability. The participant must guess whether a playing card contains a target stimulus (a color or number). The next card is displayed only after a correct response. In this way, the correct card dimension is taught. The dimension changes after a while, and the new rule must be learnt to proceed. The task terminates after 120 correct responses. Outcome variable: the arcsine transformation of the square root of the proportion of correct responses.

##### The Groton Maze Learning Test

This hidden maze task taps on multiple and more complex aspects of EF, like working memory updating, planning, and problem solving, and encompasses a notable visuospatial component. A 28-step pathway is hidden among 100 possible locations in a 10×10 grid of tiles on the screen. After learning the task rules in a practice grid, the participant guesses the pathway from the top left corner to the bottom right corner by clicking on one tile at a time, receiving continuous feedback. The task is repeated five times, with the same pathway. Outcome variable: the number of errors from the first test round (i.e., the round after the first learning trial).

##### The Continuous Paired Associate Learning Test

This task is based on the visual paired associate learning paradigm. It measures the ability to encode sets of associations between spatial locations and simple patterns, so that exposure to one aspect of the information stimulates recall of the other. First, the location of eight different figures hidden behind circles on the screen is taught. The figures are then one at a time shown at the screen’s center, and the participant is to remember behind which circle the figure is hidden during six test rounds. Outcome variable: the number of errors from the first test round (i.e., the round after the first learning trial).

##### The International Shopping List Test

This is a verbal list learning task, which taps into especially verbal working memory updating. The task has three rounds, where a shopping list of 12 items is read out loud, and the participant is asked to recall the items. Outcome variable: the number of correct responses from the first round.

#### Covariates

Age, verbal intelligence, and educational attainment are known to be associated with EF capacity (Zelazo et al., 2004; Friedman et al., 2006; Deary and Johnson, 2010). They were therefore considered as candidates for covariates in the current study. Verbal intelligence was assessed with the Wechsler Adult Intelligence Scale, fourth edition, Verbal Comprehension Index (WAIS-IV VCI; Wechsler, 2012). The WAIS-IV is a broadly used intelligence test for adults, and the VCI is derived from the verbal subtests similarities, information, and vocabulary. The VCI is calculated using scaled scores, which are based on age-specific norms.

### Analytic Approach

All analyses were performed with SPSS (version 26). All variables were evaluated for normality. Mean values and standard deviations were calculated. The EPDS, SCL-90, and AIS mean scores were compared to recommended clinical cutoff scores. The WAIS-IV subtest scaled scores and the VCI scores were calculated using the Finnish norms (Wechsler, 2012). The Cogstate tasks with available normative data (i.e., TWOB, GML, CPAL, and ISL) were compared with unpublished normative data for healthy adults (Cogstate, 2014). The Cogstate completion pass rate and integrity pass rate were calculated. Some mothers had encountered Cogstate during a prior study visit. Practice effects were controlled for by comparing the first-time participants’ (*n*=118) results with re-tested participants’ (*n*=32) results using the Mann–Whitney *U* test. Bivariate correlations between the covariates, the independent variables, and the dependent variable were calculated.

We examined to what degree psychological distress, which is common in early parenthood, is associated with maternal EF variation with a hierarchical multiple regression analysis. Of the control variables, only education level correlated significantly with maternal EF. Thus, the other control variables were omitted from the regression analyses. In Step 1, we added education level. We then added symptoms of depression/anxiety/insomnia and poor couple relationship adjustment as continuous variables in Step 2, in order to study both the single and the additive effects of these psychological distress symptoms on maternal EF level.

To capture potential variation in the psychological distress/EF association depending on whether the symptoms crossed cutoffs indicating clinically elevated levels, we complemented our examination of the psychological distress measures as continuous variables (spanning subclinical to clinically elevated symptom levels) with a dichotomous comparison between mothers reporting/not reporting clinically elevated levels. We started by dichotomizing our continuous distress variables, i.e., symptoms of depression/anxiety/insomnia and poor couple relationship adjustment. All values below the clinical cutoffs were recoded as 0, and all values above the cutoffs were recoded as 1 (cutoff values from Schmitz et al., 2000; Soldatos et al., 2003; Turliuc and Muraru, 2013; Smith-Nielsen et al., 2018). We ran four separate hierarchical multiple regression analyses, in which education level was added as a control variable in Step 1, and the four dichotomized distress measures were added one-by-one in Step 2.

We also examined whether the cumulative amount of clinically elevated distress levels in different domains (i.e., depression, anxiety, insomnia, and couple relationship adjustment) was associated with maternal EF level. The four dichotomized distress domains were combined into a sum variable, describing how many domains crossing the cutoffs for clinically elevated levels the participants reported simultaneously. We then used a hierarchical multiple regression analysis to examine whether the amount of concurrent elevated distress levels in different domains was associated with EF variation. In Step 1, we added education level as a control variable. In Step 2, we added the number of domains with clinically elevated levels (as a continuous variable). Finally, the association between the number of domains with clinically elevated levels and maternal EF level was visualized in a scatterplot with a fitted regression line.

## Results

### Descriptive Statistics

The means, standard deviations, and ranges of the questionnaires and cognitive tests are presented in Table 1. Few mothers reported high depression/anxiety levels (as can be expected in a general population sample), but a larger proportion reported clinically elevated levels of insomnia and couple relationship distress. Table 2 depicts the participants’ distress measure results grouped according to cutoff levels, as well as the percentage of participants reporting clinically elevated symptoms within 0–4 concurrent domains. As again expected in a general population sample, most participants reported clinically elevated levels in either zero or one domain, while a small subgroup reported symptoms crossing clinical cutoffs in multiple domains.

**Table 1:** Mean values, standard deviations, and ranges for questionnaires and cognitive tests.

Variable	Mean	*SD*	Range
EPDS	3.63	3.73	0–18
SCL-90	2.77	3.40	0–16
AIS	5.57	3.35	0–18
RDAS	49.67	8.01	21–66
Cogstate			
TWOB	1.30	0.13	1.00–1.57
SETS	1.19	0.11	0.92–1.33
GML	8.64	3.33	1–19
CPAL	12.44	8.41	0–40
ISL	7.87	1.50	4–12
WAIS-IV VCI	102.72	15.33	66–136

*EPDS, Edinburgh Postnatal Depression Scale; SCL-90, Symptom Checklist-90 anxiety subscale; AIS, Athens Insomnia Scale; RDAS, Revised Dyadic Adjustment Scale; TWOB, Two Back Test; SETS, Set-Shifting Test; GML, Groton Maze Learning Test; CPAL, Continuous Paired Associate Learning Test; ISL, International Shopping List Test; WAIS-IV VCI, Wechsler Adult Intelligence Scale-IV Verbal Comprehension Index*.

**Table 2:** Frequency of clinically elevated symptom domains in the study sample.

Symptom measure	No symptoms	Subclinical symptoms		Clinically elevated symptoms	
EPDS	22.0%	70.7%		7.3%	
SCL-90	33.3%	55.4%		11.3%	
AIS	3.3%	48.0%		48.7%	
RDAS	0.0%	63.3%		36.7%	
Number of symptom domains with concurrent clinically elevated levels	0	1	2	3	4
	38.0%	32.0%	20.0%	8.0%	2.0%

*EPDS, Edinburgh Postnatal Depression Scale; SCL-90, Symptom Checklist-90 anxiety subscale; AIS, Athens Insomnia Scale; and RDAS, Revised Dyadic Adjustment Scale*.

The TWOB and GML mean sample scores were within the normal range (±1 *SD*) of Cogstate normative data for the age groups 18–34/35–49years. The ISL mean sample scores were within the normal range for the 18–34-year age group, and slightly better than for the 35–49-year age group. The CPAL mean sample scores reflected more errors than expected based on the norms; however, the CPAL normative sample size is very small (18–34years *N*=62, 35–49years *N*=9) and should thus be referred to with caution. The Cogstate integrity pass rate was 100% for all tasks except for TWOB; seven participants’ TWOB results were excluded due to an insufficient pass rate. The completion rate was 100% for all tasks except for SETS; one participant’s SETS result was incomplete and excluded. The mothers tested with Cogstate for the first time vs. the re-tested mothers did not have significantly different results (*U*-tests, *p*=0.35–0.95). The participants’ WAIS-IV VCI results were representative of the general Finnish population (normative *M*=100, *SD*=15). Nine participants had not completed the WAIS-IV VCI tasks due to time restrictions.

### Correlational Results

As can be seen in Table 3, the correlations between the psychological distress measures as continuous variables and EFs were all in the expected direction (i.e., more symptoms – lower EFs), but these associations were weak. All symptom domains correlated with each other on a significant level, so that a higher number of symptoms assessed with different measures covaried with each other. Of the control variables, only education level correlated significantly (and positively) with maternal EFs. Hence, education level was utilized as a control variable in the subsequent regression analyses.

**Table 3:** Bivariate correlations between variables.

S. No.	Variable	1	2	3	4	5	6	7	8
1.	EPDS^b^	1							
2.	SCL-90^b^	0.75^**^	1						
3.	AIS^b^	0.51^**^	0.42^**^	1					
4.	RDAS^b^	0.21^**^	0.27^**^	0.33^**^	1				
5.	Age	0.08	−0.04	0.14	0.15	1			
6.	Education level^a^	0.17^*^	0.03	0.03	−0.01	0.30^**^	1		
7.	WAIS-IV VCI^a^	0.15	0.02	−0.07	0.12	0.31^**^	0.40^**^	1	
8.	EF^a^	−0.08	−0.13	−0.15	−0.17^*^	−0.12	0.22^*^	0.15	1

** *Correlation is significant at the 0.01 level (two tailed)*.

* *Correlation is significant at the 0.05 level (two tailed). Pearson correlations were calculated for all variables except for education level, and for which, Spearman correlations were calculated*.

a *Higher score=more advantageous*.

b *Lower score=more advantageous*.

*EPDS, Edinburgh Postnatal Depression Scale; SCL-90, Symptom Checklist 90 Anxiety Subscale; AIS, Athens Insomnia Scale; RDAS, Revised Dyadic Adjustment Scale; WAIS-IV VCI, Verbal Comprehension Index Wechsler Adult Intelligence Scale-IV; and EF, Cogstate EF composite*.

### Multiple Regression Results

The first step of all the regression models was identical, including only the control variable education level, which accounted for 4% of maternal EF variation on a significant level (*R*^2^=0.04, *p*=0.01). Thus, the Step 1 results are not repeated in the subsequent results description.

The results of the regression analysis examining the associations between single/additive psychological distress domains and maternal EF variation are presented in Table 4. Contrary to our expectations, the continuous psychological distress symptoms added in Step 2, i.e., EPDS, SCL-90, AIS, and RDAS, did not have significant effects on EFs (*∆R*^2^=0.04, *p*=0.20).

**Table 4:** Associations between continuous symptoms and executive functioning.

	*R* ^2^	*R* ^2^ *∆*	*F∆*	*F∆* value of *p*	*B*	*β*	*t*	*B* value of *p*	*B*, 95.0% confidence interval	sr^2^
**Step 1** Education level	0.04	0.04	6.67	0.01						
					0.27	0.21	2.58	0.01	0.06/0.47	0.04
**Step 2**	0.08	0.04	1.52	0.20						
EPDS					0.01	0.05	0.36	0.72	−0.06/0.08	0.00
SCL-90					−0.02	−0.08	−0.67	0.50	−0.10/0.05	0.00
AIS					−0.03	−0.11	−1.12	0.27	−0.09/0.03	0.01
RDAS					−0.01	−0.11	−1.25	0.21	−0.04/0.01	0.01

*EPDS, Edinburgh Postnatal Depression Scale; SCL-90, Symptom Checklist-90 anxiety subscale; AIS, Athens Insomnia Scale; and RDAS, Revised Dyadic Adjustment Scale*.

As can be seen in Table 5, when the psychological distress domains were dichotomized to compare symptom levels below/above their respective clinical cutoffs, the symptom domains were not independently associated with EF variation (EPDS: *∆R*^2^=0.00, *p*=0.46; SCL-90 *∆R*^2^=0.01, *p*=0.40; AIS: *∆R*^2^=0.02, *p*=0.11; RDAS *∆R*^2^=0.02, *p*=0.08). However, there was a slight difference between the symptom domains: The associations with EFs were clearly non-significant for depression and anxiety, while the associations came close to significance for insomnia and poor couple relationship adjustment.

**Table 5:** Associations between single elevated symptom domains and executive functioning.

	*R* ^2^	*R* ^2^ *∆*	*F∆*	*F∆* value of *p*	*B*	*β*	*t*	*B* value of *p*	*B*, 95.0% confidence interval	sr^2^
**Depression**										
**Step 1** Education level	0.04	0.04	6.67	0.01	0.27	0.21	2.58	0.01	0.06/0.47	0.04
**Step 2** EPDS	0.05	0.00	0.56	0.46	−0.23	−0.06	−0.75	0.46	−0.84/0.38	0.00
**Anxiety**										
**Step 1** Education level	0.04	0.04	6.67	0.01	0.27	0.21	2.58	0.01	0.06/0.47	0.04
**Step 2** SCL-90	0.05	0.01	0.72	0.40	−0.22	−0.07	−0.85	0.40	−0.71/0.29	0.00
**Insomnia**										
**Step 1** Education level	0.04	0.04	6.67	0.01	0.27	0.21	2.58	0.01	0.06/0.47	0.04
**Step 2** AIS	0.06	0.02	2.63	0.11	−0.26	−0.13	−1.62	0.11	−0.57/−0.06	0.02
**Poor couple relationship adjustment**										
**Step 1** Education level	0.04	0.04	6.67	0.01	0.27	0.21	2.58	0.01	0.06/0.47	0.04
**Step 2** RDAS	0.06	0.02	3.06	0.08	−0.29	−0.14	−1.75	0.08	−0.62/0.04	0.02

*The dichotomized psychological distress measures were split according to cutoffs, separating between participants reporting/not reporting clinically elevated symptom levels. EPDS, Edinburgh Postnatal Depression Scale; SCL-90, Symptom Checklist-90 anxiety subscale; AIS, Athens Insomnia Scale; and RDAS, Revised Dyadic Adjustment Scale*.

Next, the association between the cumulative number of clinically elevated psychological distress domains and maternal EF level was studied (Table 6). In line with our expectations, the number of domains with clinically elevated levels was significantly associated with the participants’ EF level, so that the number of domains predicted 3% of maternal EF variation (*∆R*^2^=0.03, *p*=0.04).

**Table 6:** Association between number of elevated symptom domains and executive functioning.

	*R* ^2^	*R* ^2^ *∆*	*F∆*	*F∆* value of *p*	*B*	*β*	*t*	*B* value of *p*	*B*, 95.0% confidence interval	sr^2^
**Step 1** Education level	0.04	0.04	6.67	0.01	0.27	0.21	2.58	0.01	0.06/0.47	0.04
**Step 2** Symptom nr.	0.07	0.03	4.19	0.04	−0.16	−0.16	−2.05	0.04	−0.31/−0.01	0.03

*Symptom nr. refers to the number of psychological distress domains (i.e., depression, anxiety, insomnia, and poor couple relationship adjustment), which cross-cutoff values for clinically elevated symptom levels*.

**Figure 1:**
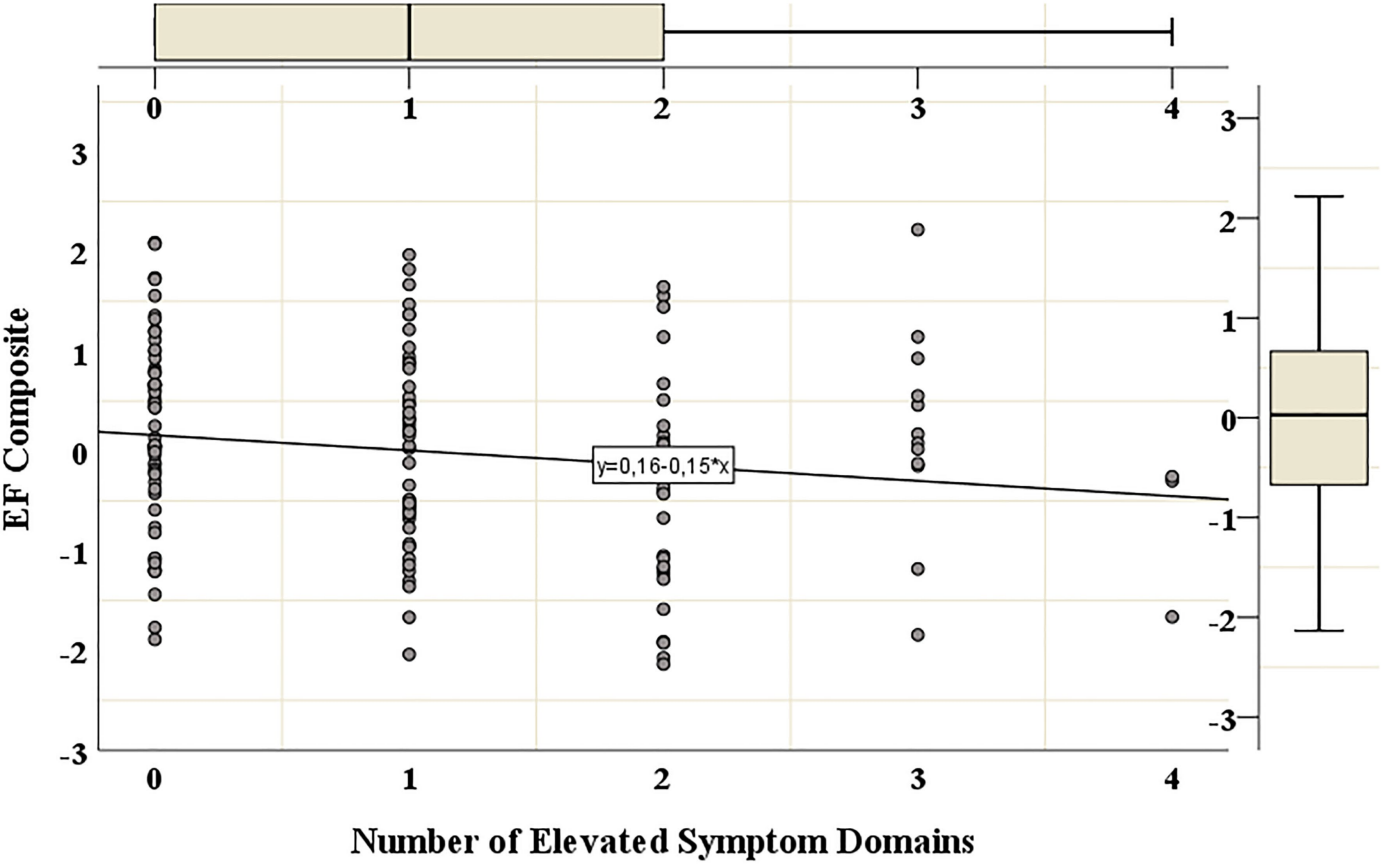
Executive functioning grouped according to number of elevated symptom domains. The scatterplot with a fitted regression line shows the distribution of the participants’ EF levels, as grouped according to the number of domains (i.e., depression, anxiety, insomnia, and poor couple relationship adjustment) with clinically elevated symptom levels. EFs were measured with a composite score based on five Cogstate tasks, a higher value indicates better EFs. The EF composite’s mean was set to zero through standardization. The values on the vertical axis refer to standard deviations from the EF mean value. Psychological distress was measured with self-report questionnaires (the Edinburgh Postnatal Depression Scale, the Anxiety Subscale from The Symptom Checklist-90, the Athens Insomnia Scale, and the Revised Dyadic Adjustment Scale). The number of elevated symptom domains refers to the number of concurrent psychological distress domains which cross-cutoff values for clinically elevated symptom levels.

The scatterplot with a fitted regression line in Figure 1 exhibits a trend showing lower maternal EF level as the number of simultaneous clinically elevated psychological distress domains increases from zero to two. This pattern is not as clear for participants reporting clinically elevated levels within more than two domains. However, as these group sizes are small (12 participants reported clinically elevated levels in three domains, while only three reported elevated levels in four domains), their results should be interpreted with caution. For groups this small, single extreme values can largely impact the distribution. For example, as depicted in Figure 1, the group reporting elevated psychological distress levels in three domains include one participant with a particularly high EF level.

## Discussion

We examined the associations between psychological distress commonly experienced in early parenthood and maternal EFs in a general population sample drawn from a Finnish birth cohort (Karlsson et al., 2018). Higher levels of self-reported maternal depression/anxiety/insomnia symptoms and poorer couple relationship quality were expected to be associated with lower maternal EF levels at 2.5years postpartum. As exposure to multiple risk factors predicts more severe adverse consequences in comparison with singular risk factor exposure (Evans et al., 2013), we further expected to find a cumulative effect, so that the psychological distress/EF associations would be stronger when concurrently considering multiple indicators. The results provided partial support for our hypotheses. Higher symptom levels were related to poorer EFs, but this association was statistically significant only for the number of symptoms crossing clinical cutoffs. The psychological distress/EF associations were not significant when examining separately their additive effects as continuous variables, nor when examining the effects of single, dichotomized symptom scores (which compared mothers reporting symptoms above/below clinical cutoff scores).

The significant negative association between the number of psychological distress domains with clinically elevated levels and EF performance can have important implications, also given the fact that in the present correlative approach, the links between psychological distress and EFs can be bi-directional. Prior research has primarily indicated that psychological distress like depression, anxiety, and insomnia precedes EF impairments (Castaneda et al., 2008; Snyder, 2013; Krause et al., 2017; Ballesio et al., 2019a). However, some studies also point to the possibility that lower EFs could precede psychological distress. EF capacity can influence the different components of stress regulation, i.e., exposure to stress, stress reactivity, and recovery from stress (Williams et al., 2009). Snyder et al. (2015) comment that although there is a wealth of evidence linking psychopathologies like depression and anxiety to poor EF task performance, it is unclear whether EF deficits are a consequence of psychopathology or are a causal risk factor for developing psychopathology. Similarly, in an interesting pilot study, Ballesio et al. (2019b) found that in a small sample of adult insomniacs, poorer EF partly predicted rumination about the negative consequences of insomnia. As rumination about lack of sleep plays a central role in the maintenance of insomnia, this finding suggests that adult EF capacity could not only be negatively affected by insomnia, but might also influence the development and maintenance of insomnia. Furthermore, adults who have had significant Attention-Deficit/Hyperactivity Disorder symptomatology (for which impaired EFs are a central feature) since childhood experience poorer couple relationship adjustment compared to healthy controls (Eakin et al., 2004), indicating that EFs could influence couple relationship quality. To summarize, experiencing clinically elevated symptom levels in multiple concurrent psychological distress domains could have a cumulative, negative influence on maternal EF capacity in early parenthood. Alternatively, mothers with poorer EFs could be more vulnerable to the development of psychological distress in several simultaneous domains during early parenthood. As these processes are not mutually exclusive, both could hold true for different individuals or for the same individuals at different times. Therefore, bi-directional links between psychological distress and EFs are relevant to consider within healthcare settings and future research on the components of parenting.

If clinically elevated symptom levels in multiple concurrent domains deplete maternal EFs during early parenthood, then interventions which relieve these symptoms are also likely to improve EFs, allowing mothers to make optimal use of their EF capacity while caring for their child. The distress domains examined in this study are generally treated with psychosocial interventions that focus on mood/stress regulation in the context of early parenthood. If lower EFs alternatively increase the vulnerability for mothers to develop clinically elevated psychological distress levels in multiple domains during early parenthood, then offering mothers with poorer EFs interventions that support their EF capacity could reduce this vulnerability. Supportive interventions for adults (regardless of their parental status) with a low EF capacity have primarily been developed within the ADHD-framework, as EF deficits are a central feature of ADHD. As described by Johnston et al. (2012), both pharmacotherapy and psychosocial treatments are known to reduce ADHD symptoms and functional impairments in interpersonal and work domains. However, very little is known about suitable interventions for adults with a low EF capacity (regardless of their ADHD status) in the context of parenting. Drawing on knowledge about evidence-based interventions for ADHD, low EF parents are likely to benefit from instruction in organizational and planning skills relevant to parenting, skill-based practice to support the development of new parenting habits, and modifying settings in which parenting is practiced so as to prompt, cue, and otherwise elicit appropriate parenting behavior (Johnston et al., 2012). It is noteworthy that even if the directionality of the association between clinically elevated psychological distress levels in several domains and lower EFs is disregarded, our results hold important implications. Low maternal EFs as well as maternal psychological distress like depression/poor couple relationship quality are linked to adverse child outcomes (Reid and Crisafulli, 1990; Bridgett et al., 2015; Crandall et al., 2015; Hahn-Holbrook et al., 2018), and as our findings suggest that maternal EFs and cumulative clinically significant psychological distress are associated, healthcare providers encountering mothers with one of these risk factors for adverse child outcomes are well advised to consider whether other risk factors also are present.

Our results also indicate that future research on the links between psychological distress and parental EFs is warranted. In particular, repeated measurements with larger study populations are called for, allowing for a temporal disentanglement of the potential causal relationships between psychological distress and EFs. Next steps would include relating the parental psychological distress/EF links to parenting behaviors, as well as broadening the focus from mothers to fathers. It would also be relevant to explore whether specific EF subcomponents (working memory, set-shifting, and inhibition) might be differently associated with psychological distress levels during early parenthood. Håkansson et al. (2019) found working memory to be a unique contributor to variance in psychological distress, while cognitive flexibility contributed uniquely to variance in parental stress among mothers with a substance abuse disorder during early parenthood, suggesting that focusing on the subcomponents of EFs might be warranted in the context of parental psychological distress during early parenthood.

If replicated, the null finding that continuous single and additive symptom scores were not associated with lower EFs can be seen as reassuring. It indicates that mothers during early parenthood who experience just a few symptoms of depression/anxiety/insomnia or poor couple relationship adjustment are not at great risk of a depleted EF capacity. As single psychological distress domains crossing clinical cutoff scores were not significantly associated with EFs, it seems like mothers experiencing clinically elevated levels in only one domain are not likely to have poorer EFs. Interestingly, this suggests that mothers experiencing high psychological distress symptoms in several domains might benefit from interventions relieving a part of their burden, so that their EF capacity could recover even when some clinically elevated distress domain would still remain.

### Caveats, Limitations, and Strengths

There are a number of caveats and limitations in the current study. First, it is important to consider the sample characteristics of our population-based study when interpreting the results. The current study did not include a disadvantaged sample – the participants were fairly highly educated, had a normative level of verbal intelligence, and reported few symptoms of depression/anxiety. Thus, the results should not be generalized to more disadvantaged populations. It is possible that lower psychological distress levels or single clinically elevated psychological distress domains could have significant associations with EFs in other samples with a broader range of stress exposures and symptom levels.

Second, the effect size of the association between the number of domains with clinically elevated symptom levels and EFs might seem modest, predicting only 3% of EF variation. However, EFs are complex functions that are influenced by a multitude of factors, such as genetic factors and academic training effects (Deary and Johnson, 2010; Friedman and Miyake, 2017). It would thus be surprising if the studied symptoms had a very strong association with maternal EFs, as they are only a handful of variables among many others influencing these functions. By comparison, education level, which is known to be robustly linked to adult EF level, accounted for 4% of our sample’s EF variation.

Third, regarding the covariates of age and verbal intelligence, these had surprisingly low correlations with EF, considering that these variables are known to be robustly interconnected (Zelazo et al., 2004; Friedman et al., 2006). As described in Nordenswan et al. (2020), the weak age/EF correlation is likely due to the sample’s fairly narrow age variation. The weak association between verbal intelligence and our EF composite score is likely due the inclusion of primarily non-verbal tasks in the Cogstate EF composite (Nordenswan et al., 2020).

Fourth, EFs were assessed with laboratory tasks, which are more reliable than questionnaires, and considered a gold standard. However, the ability to use one’s EFs can differ from a structured laboratory environment to real-life situations. The combination of several EF tasks into a composite score can be seen as a strength, as it minimizes variability due to measurement errors. A central measurement-related limitation is that the five tasks included in our EF composite primarily measure working memory and set-shifting, and a standard measure of response inhibition was not included. Furthermore, the EF tasks encompass notable elements of learning and could also be labeled “learning/EF-tasks” (Nordenswan et al., 2020). In addition, EFs were only measured once. Repeated assessments would allow for more nuanced analyses of potential fluctuations in EF capacity following differing stress levels. Still, given how time intensive EF task data collection is, we view the study that we conducted as an essential first step that can pave the way for future longitudinal investigations.

Finally, turning to other measurement limitations, psychological distress domains were measured with self-report questionnaires. This allows for potential reporting biases; e.g., partner or observer ratings would likely result in somewhat differing assessments of the participants’ depression/anxiety/insomnia symptoms and couple relationship quality. However, the utilized self-reports do encompass an important aspect of ecological validity with regard to healthcare settings, as healthcare providers assessing maternal psychological distress during early parenthood often rely on the same or very similar questionnaires to those that we used. In addition, couple relationship adjustment was assessed 6months before the other variables. It is thus possible that some participants no longer had a partner during the latter assessment or that couple relationship adjustment had changed between assessment points. However, this is probably not a major concern, given that couple relationships often change slowly.

## Conclusion

Our results indicate that for general population mothers with very young children, milder psychological distress levels as well as clinically elevated symptom levels within only one of the studied domains (i.e., depression, anxiety, insomnia, and poor couple relationship adjustment) were not significantly associated with EFs. However, the expected negative association with EF was found when the overall number of clinically elevated distress domains was examined, and this warrants further attention as it suggests a cumulative, depleting effect on maternal EF capacity. It is also possible that lower maternal EF capacity increases the vulnerability for several simultaneous clinically elevated psychological distress domains. We recommend that policy makers and healthcare providers recognize potential associations between maternal cumulative psychological distress and poorer maternal EFs during early parenthood. Suitable interventions that relieve psychological distress could allow mothers to make optimal use of their EF capacity while caring for their child, and EF-supporting interventions could decrease the risk for clinically elevated psychological distress levels within several concurrent domains. Future studies including repeated measurements are needed to shed light on potential bi-directional causal links, with a particular need for studies that examine these processes in disadvantaged populations.

## Data Availability Statement

The datasets presented in this article are not readily available because the medical faculty at the University of Turku has strict legal data sharing rules. The anonymized dataset is available upon request. Requests to access the datasets should be directed to statistician Juho Pelto, juho.pelto@utu.fi.

## Ethics Statement

The studies involving human participants were reviewed and approved by the Joint Ethics Committee of Turku University Hospital and University of Turku. The participants provided their written informed consent to participate in this study.

## Author Contributions

EN: data collection, methodology, and writing – original draft. KD-D, MK, and ML: supervision, methodology, and writing – review and editing. E-LK: data collection, methodology, and writing – review and editing. JP: methodology and writing – review and editing. HK and LK: funding acquisition, methodology, and writing – review and editing. RK: supervision, funding acquisition, methodology, and writing – review and editing. All authors contributed to the article and approved the submitted version.

## Funding

This work was supported by the Academy of Finland [grant numbers 253270, 134950, 286829, 308252 (RK), and 325292 Profi 5 (LK)], the Jane and Aatos Erkko Foundation, the Signe and Ane Gyllenberg Foundation (LK and RK), the State Research Funding of the Turku University Hospital (LK and RK), the Finnish Cultural Foundation (E-LK and RK), the Victoria Foundation (EN), and the Agneta and Carl-Erik Olin Foundation (EN). Funds for the open access publication fee were received from the Åbo Akademi University APC pool funding. The funders had no role in study design, data collection and analysis, decision to publish, or preparation of the manuscript.

## Conflict of Interest

The authors declare that the research was conducted in the absence of any commercial or financial relationships that could be construed as a potential conflict of interest.

## Publisher’s Note

All claims expressed in this article are solely those of the authors and do not necessarily represent those of their affiliated organizations, or those of the publisher, the editors and the reviewers. Any product that may be evaluated in this article, or claim that may be made by its manufacturer, is not guaranteed or endorsed by the publisher.
